# Pediatric Tonsillar Synovial Sarcoma- Very Rare Localization: A Case Report and Review of the Literature

**DOI:** 10.5146/tjpath.2018.01449

**Published:** 2020-01-15

**Authors:** Koray Yalçın, Gülen Tüysüz, Mine Genç, İrem Hicran Özbudak, Alper Tunga Derin, Kamil Karaali, Alphan Küpesiz, Elif Güler

**Affiliations:** Department of Pediatric Hematology and Oncology, Akdeniz University Medicine Faculty, Antalya, Turkey; Department of Radiation Oncology, Akdeniz University Medicine Faculty, Antalya, Turkey; Department of Pathology, Akdeniz University Medicine Faculty, Antalya, Turkey; Department of Nose Throat Ear Surgery, Akdeniz University Medicine Faculty, Antalya, Turkey; Department of Radiology, Akdeniz University Medicine Faculty, Antalya, Turkey; Department of Pediatric Hematology and Oncology, Akdeniz University Medicine Faculty, ANTALYA, TURKEY

**Keywords:** Synovial Sarcoma, Tonsillar neoplasm, Pediatric tumor, Dysphagia, Snoring

## Abstract

Tonsillar synovial sarcoma is an extremely rare entity and only 9 adult patients have been reported up to now. Here, we describe the first pediatric tonsillar synovial sarcoma of the literature in a patient who presented with a 2-month history of dysphagia and snoring. Clinical and radiological examinations showed that the tumor arose from the right palatine tonsil and narrowed the parapharyngeal space. An incisional biopsy from the palatine tonsil revealed the diagnosis of synovial sarcoma. The patient has underwent total tonsillectomy and received radiotherapy and chemotherapy because of the positive surgical margins. The patient is clinically in good condition and free of tumor 30 months after the initial diagnosis. We achieved a long-term complete remission with a combination of surgery, radiotherapy and chemotherapy in our case. Tonsillar synovial sarcoma should be kept in mind while dealing with tonsillar masses. We can conclude that a multidisciplinary approach is warranted while treating synovial sarcoma with this localization.

## INTRODUCTION

Synovial sarcoma (SS) is the most common soft tissue sarcoma after rhabdomyosarcoma in the pediatric population. The annual incidence rate is 0.5-07 /million in children and adolescents younger than 20 years of age (1,2). Synovial sarcoma primarily arises from deep soft tissues of the extremities, usually from the lower extremities followed by the upper extremities, trunk, and retroperitoneal/abdominal region. Head and neck localization is rare with a percentage of only 3-10% of all SS cases (3,4). The most frequent areas in the head and neck region include the hypopharynx, followed by the parapharyngeal space and post pharyngeal area. Primary SS of the palatine tonsil is extremely rare and only 9 adult cases have been reported so far (5–11). As far as we are aware, we report the first pediatric case of tonsillar SS in the literature. Because of the unusual localization, the management and treatment of tonsillar SS is based on case reports (5–11). It is known that both the treatment and prognosis of SS differs among children and adults (2,12). We therefore believe this report may contribute to the existing literature with its rare location and treatment approach.

## CASE REPORT

**Figure 1:**
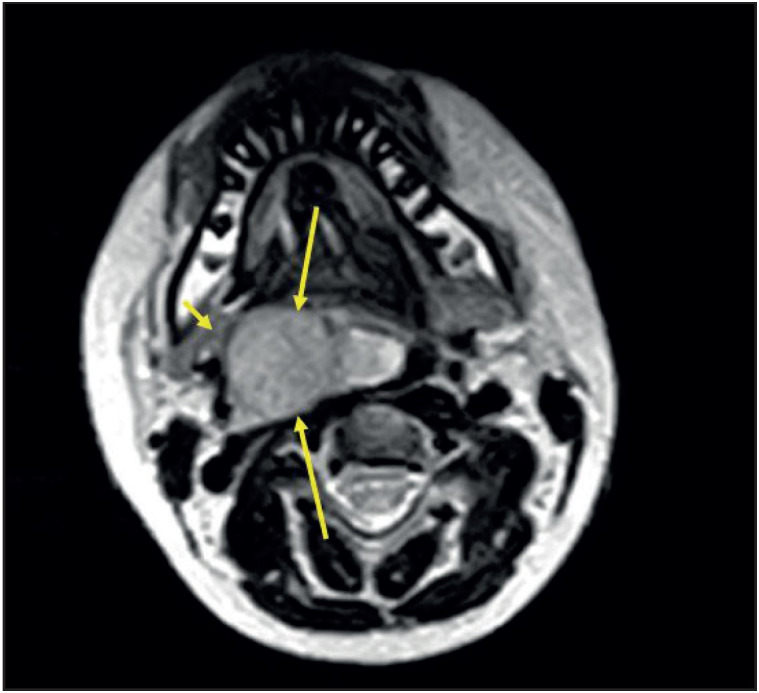
T2-weighted axial MR image shows hyperintense mass lesion within the right parapharyngeal area (long arrows). Maximum diameters in this plane are 24 x 38 mm. The mass is displacing the right submandibular gland (short arrow) but there is no invasion.

**Figure 2:**
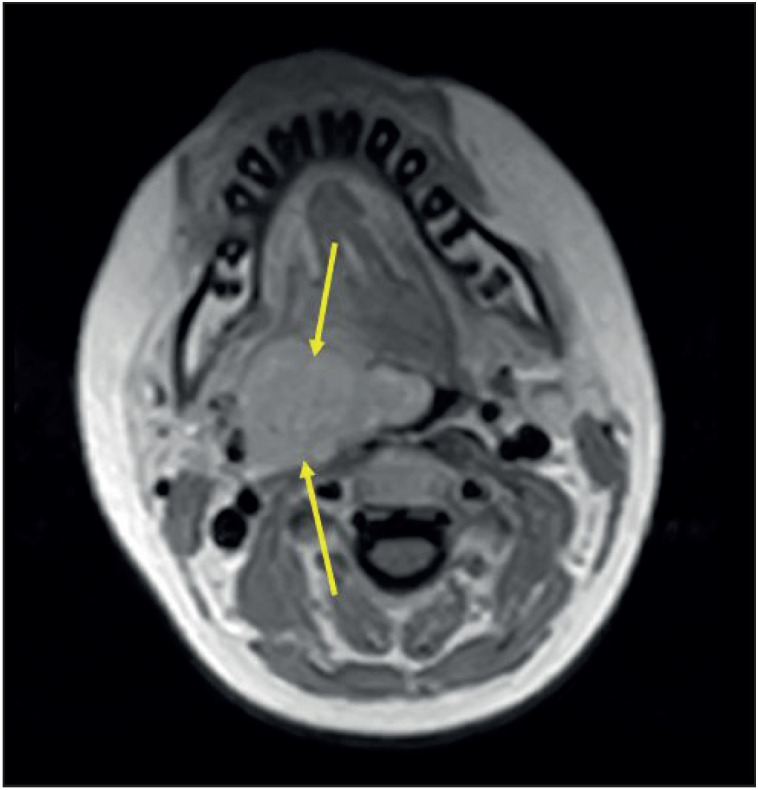
T1-weighted post contrast image shows diffuse homogeneous enhancement of the mass lesion (arrows).

**Figure 3:**
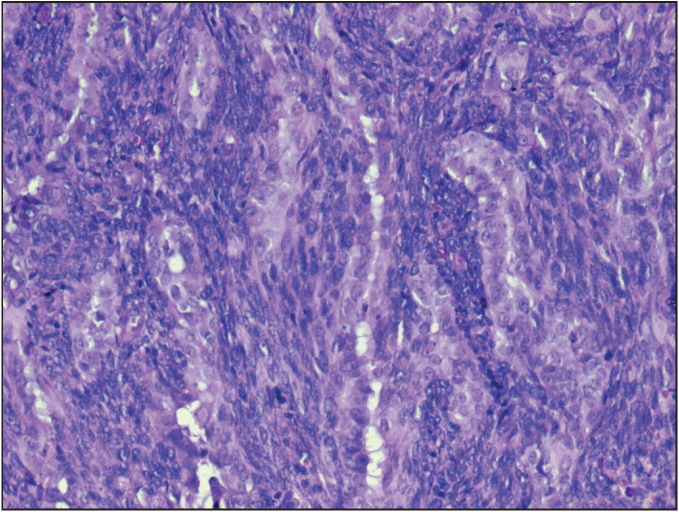
Biphasic architecture of neoplasm composed of spindle cells and epithelial cells (H&E; 200).

**Figure 4:**
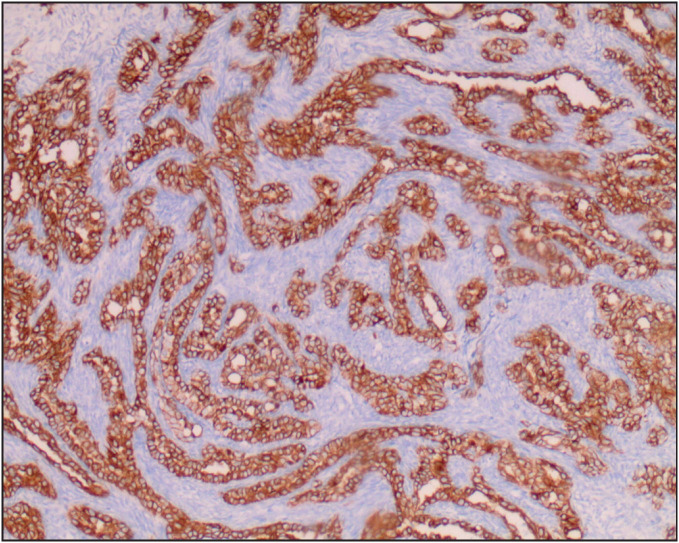
CK7 positivity in neoplastic epithelial cells (IHC; x100).

A 13-year-old boy presented with a 2-month history of dysphagia and snoring. His physical examination revealed an ulcerative, green colored mass in the right tonsil which narrowed the oropharynx. Contrast-enhanced magnetic resonance imaging (MRI) revealed a 50x47x45 mm solitary mass originating from the right lateral oropharynx, extending to the hypopharynx and narrowing the oropharyngeal lumen irregularly (Figure 1 Figure 2). The Positron Emission Tomography (PET) scan showed metabolic activity (SUV max: 9.3) in the right tonsillar area. There was no evidence of distant metastases or regional lymph node involvement in the PET scan. To make a diagnosis, an incisional biopsy was performed from the right tonsil and the histopathological examination of the specimen revealed the presence of a biphasic SS. Immunohistochemical examination for PanCK, CK19, CK18, CK7 revealed positive staining in the epithelial component and staining for vimentin revealed positive staining in the mesenchymal component. The Hematoxylin-Eosin staining of tumor is illustrated in Figure 3 and the CK7 immunopositivity of the epithelial cells is illustrated in Figure 4. Muscle and neural markers were negative. The Ki67 proliferation index was 50%. The diagnosis was confirmed by additional cytogenetic analysis, which revealed the presence of SYT-SSX1, a specific fusion gene for synovial sarcoma in the tumor cells.

The patient was staged according to IRS staging as clinical group 3 and chemotherapy was initiated with vincristine (1,5 mg/m2; weekly), ifosfamide (3 g/m2; 1 in 3 weeks), and doxorubicin (30 mg/m2; 1 in 3 weeks) according to the treatment schema for the Pediatric Oncology Group trial 9553 (13). The tumor remarkably regressed on the 10th week of treatment. The patient had undergone total tumor excision but a total of 6000 cGy conventional radiotherapy was administered to the primary tumor site in 30 fractions due to the positive surgical margins. Chemotherapy was completed at 23 weeks according to the protocol. At the last follow-up 30 months after the initial diagnosis, the patient was still in complete remission with a 100 Karnofsky performance score.

## DISCUSSION

Synovial sarcoma is often associated with joints such as knee, but it can also arise from pluripotential mesenchymal stromal cells anywhere in the body (8). Histologically this tumor is classified based on epithelial and spindle cell components in two subtypes; monophasic and biphasic. Synovial Sarcoma rarely occurs in the head and neck area but the parapharyngeal space is the most commonly involved location for this region when it does. The monophasic subtype of SS is the most common histological pattern in the head and neck area (3,14,15).

Primary SS of the tonsil is extremely rare and only nine cases have been reported in the English literature up to now (5–11) (Table 1). All of these patients are adults (> 18 ages) with male gender. Our case is the youngest case and the first pediatric patient. Previous studies reported the dominancy of monophasic spindle cell variant for head and neck SS (3,14,15) but 7 of the reported tonsillar SS patients and ours had the biphasic subtype.

**Table 1:** The review of previous patients with Tonsillar Synovial Sarcoma.

**References**	**Age (year)**	**Gender**	**Localization** **(palatine tonsil)**	**Histopathology**	**Genetic Study**	**Surgery**	**RT**	**CT**	**Recurrence**	**Salvage** **treatment**	**Status**
(8)	21	Male	Left	Biphasic	NA	Complete surgical resection	+	+	-		At 10 years, CR
(10)	63	Male	Right	Monophasic	SYT/SSX1	Complete surgical resection	-	-	-	-	At 1 year CR, lost to flollow up
(11)	31	Male	Left	Biphasic	SYT/SSX1	Complete surgical resection	-	-	-	-	At 4 years, CR
(12)	19	Male	Right	Biphasic	SYT/SSX1	S		-	At 16 months, servical metastases	Complet surgery	At 2 years,10 months CR
(13)	25	Male	Right	Biphasic	NA	S	-	-	-	-	CR
(14)	35	Male	Right	Biphasic	NA	NA	NA	NA	NA	NA	NA
(14)	34	Male	Left	Biphasic	NA	Surgery	+	-	-	-	At 3 years, CR
(15)	23	Male	Right	Monophasic	NA	Incomplete surgery	60 Gy	CYVADICX6	At 18 months, local recurrence	Debulking surgery + CT	At 3 years DD
(15)	26	Male	Right	Biphasic	NA	Incomplete surgery	60 Gy	CYVADICX6	At 22 months, local recurrence	Debulking surgery + CT	At 3 years alive with disease
*Present case*	13	Male	Right	Biphasic	SYT/SSX1	BX, delayed surgery	60 Gy	VID	-	-	At 3 years, CR

**S:** Surgery, **RT:** Radiotherapy, **CT:** Chemotherapy, **bx:** Biopsy, **CR:** Complete remission, **NR:** Non-remision, **DD:** Died of disease, **NA:** No data available

More than 90% of all SS show a reciprocal translocation, t (X: 18) (p11:q11), with a resultant fusion gene of SYT and one of three SSX genes. It is considered a molecular hallmark for the diagnosis of SS in difficult cases (16). Three of the cases previously reported and ours had this diagnostic translocation.

Complete surgical excision with a wide margin is the mainstay of the treatment in SS. In cases with positive surgical margins, a high incidence of local recurrence (60%-90%) within 2 years has been reported (17). Radiotherapy is strongly recommended to prevent local recurrences in patients with microscopic residual disease (1–3,17). The role of chemotherapy in adults and children with SS remains controversial. Synovial sarcoma in adults generally has been considered a less chemosensitive tumor and patients have been treated with surgery and radiotherapy (18,19). In contrast, synovial sarcoma in pediatric patients is considered to be more chemosensitive than in adults and these patients have been treated according to rhabdomyosarcoma chemotherapy protocols. The chemotherapy response rate has been estimated to be 60-65% in children. Many drugs are active on this tumor, but the most commonly used regimens include doxorubicin and/or ifosfamide (2,20,21).

It has been shown that a large tumor size (≥5 cm in diameter), presence of distant metastasis, gross residual disease, and omission of radiotherapy are all significantly associated with poor survival (1). Age is described as an independent prognostic factor for survival of localized SS. The 5-year overall survival has been reported as 89% in children and 43% in adults (22).

Head and neck localization of SS is rare and the treatment approach to tonsillar SS is based on case reports. There are nine case reports in the literature (Table 1). All of the reported patients are adults and they were treated according to adult protocols. Six of the previously reported cases had initial surgery and the tumor was totally removed (5–11). Radiotherapy was applied to 2 of these patients and chemotherapy was administered to one of them. All of these patients were in complete remission within a median of 3.8 years of follow up when they were published as case reports. Two cases reported by Khademi et al. (11) had no chance for total excision because of an extended tumor. They underwent debulking surgery and then received radiotherapy and chemotherapy respectively. They both had local recurrence; one of them died because of progressive disease, and the other had progressive disease despite salvage treatment. Our case also presented with a large and extensive tumor. After biopsy, he first received neoadjuvant chemotherapy and underwent surgery when the tumor regressed. Radiotherapy was applied for microscopic residue and chemotherapy was continued up to 23 weeks according to the protocol (13).

Although there are limited data about tonsillar synovial sarcoma in the literature, it is obvious that total tumor excision is the most crucial part of the treatment. Neoadjuvant chemotherapy is recommended for all patients with SS with a head and neck localization who have tumors > 5 cm in size, extensive or recurrent disease, and high risk localization, such as the skull base or paraspinal area (3,14,15,23). Pediatric oncologists have changed the treatment of SS depending on the patient’s risk stratification. Patients with macroscopic disease, N1 tumor and axial localization (head-neck, trunk, lung-pleura, retro peritoneum) are classified as a high risk group regardless of any other clinical parameters. These patients are now more prone to be treated with neoadjuvant chemotherapy, delayed surgery and radiotherapy (20). It has however been suggested that adjuvant chemotherapy could be omitted for low-risk pediatric patients (20,24).

In conclusion, a review of the literature shows that surgery could be sufficient for low risk and totally excisable tonsillar SS cases. However, we believe that high risk patients such as our case deserve multimodal therapy. The treatment approach should include a multidisciplinary team consisting of a head and neck surgeon, radiation oncologist and pediatric oncologist to ensure full recovery in these patients.

## FUNDING

This study did not receive any specific grant from funding agencies in the public, commercial, or not-for-profit sectors.

## Conflict of Interest

The authors declare no conflict of interest.
